# Highly Sensitive and Stable Humidity Sensor Based on the Bi-Layered PVA/Graphene Flower Composite Film

**DOI:** 10.3390/nano12061026

**Published:** 2022-03-21

**Authors:** Sheik Abdur Rahman, Shenawar Ali Khan, Muhammad Muqeet Rehman, Woo-Young Kim

**Affiliations:** Faculty of Applied Energy System, Major of Electronic Engineering, Jeju National University, Jeju 63243, Korea; abdurrahman@jejunu.ac.kr (S.A.R.); shenawaralikhan@jejunu.ac.kr (S.A.K.); muqeet1988@jejunu.ac.kr (M.M.R.)

**Keywords:** bi-layered, graphene flower, humidity sensor, poly (vinyl alcohol) PVA

## Abstract

Two-dimensional (2D) materials and their composites have gained significant importance as the functional layer of various environmental sensors and nanoelectronics owing to their unique properties. This work reports for the first time a highly sensitive, fast, and stable humidity sensor based on the bi-layered active sensing area composed of graphene flower (GF) and poly (vinyl alcohol) PVA thin films for multifunctional applications. The GF/PVA humidity sensor exhibited stable impedance response over 15 days, for a relative humidity (RH) range of (40–90% RH) under ambient operating conditions. The proposed bi-layered humidity sensor also exhibited an ultra-high capacitive sensitivity response of the 29 nF/%RH at 10 kHz and fast transient response of 2 s and 3.5 s, respectively. Furthermore, the reported sensor also showed a good response towards multi-functional applications such as non-contact skin humidity and mouth breathing detection.

## 1. Introduction

Thin film-based capacitive and impedimetric sensors offer numerous benefits over optical and chemical sensors in vapor detection applications, namely low energy consumption, higher sensitivity response and selectivity, good stability, and fast response/recovery time [1,2]. In industrial, agricultural, and medical services, moisture content is a critical environmental factor. Humidity sensors have recently emerged as promising candidates for use in electronic skin (e-skin), health monitoring applications, soft robotics, and noncontact sensing [3,4,5,6]. Noncontact sensing and contactless interface localization applications can be employed with a humidity sensor array, which reduces the hazard of bacterial transmission that can occur with conventional touch displays. There is indeed a lot of research going on at present towards the development of efficient, low-cost, and simple-to-interface sensors for monitoring and controlling ambient humidity. According to some studies, wide sensing range is the most important factor, whereas others regard fast response/recovery time as the most significant characteristic of a humidity sensor. To improve the property of interest, scientists develop new sensor structures, materials, and working mechanisms. The primary goal of contemporary research is to produce capacitive and impedimetric sensors by developing new materials with desirable properties. Most research on material-based humidity sensors has been based on developing either a single material or a composite to attain the desired performance, while other research has used bi-layered sensing film structures. In these types of sensors, a large variety of material categories have already been explored and tested as humidity transducing elements, which include polymers, metal oxides, 2D materials, biomaterials, composites, carbon derivatives, etc. [7,8,9,10,11,12,13,14,15,16].

Carbon-based materials, such as graphene and carbon nanotubes (CNTs), have sparked a great deal of interest in chemical sensing because of their distinctive structural and electronic properties, such as a large surface area, compact size, high electronic mobility, and high sensitivity response to electrical interruptions from water or other gaseous molecules [17,18,19,20,21,22]. In addition, because of its exceptional thermal, mechanical, and electrical characteristics, graphene is the first and most appealing 2D material for use in a variety of applications. Graphene’s unique properties originate from its two-dimensional monolayer of carbon atoms, which create a one-atom-thick honeycomb-like structure, making it ideal for super-sensitive monitoring applications due to its huge surface-to-volume ratio [23,24,25,26,27]. There have been reports of graphene-based gas sensors with sensitivity sufficient for detecting a single gas molecule. Graphene creates pi-bonds with surrounding atoms owing to its p-orbital electrons, and the charged particles of these pi-bonds are responsible for the increased sensitivity to any environmental change, making graphene excellent for biological and chemical sensing applications [26,28,29].

Polymers are another class of material with a high sensitivity to many environmental factors. Low cost, simple synthesis, high stability, and good mechanical characteristics for flexible electronics are just a few of the major advantages of utilizing these materials [30,31,32,33,34]. Poly vinyl alcohol (PVA) was chosen as a humidity-sensing polymer because of its hygroscopic nature and various hydrophilic functional groups, which have a high affinity for water [35,36]. It also has a high level of thermal and chemical stability. This material has demonstrated promising results in pressure sensors, eco-friendly health monitoring devices, and temperature-sensing applications [37,38,39]. When water vapor is adsorbed onto PVA, the conductivity value changes, and with increasing humidity, there is more hydrogen bonding between water vapors and the OH groups in PVA. On the other hand, PVA has a high intrinsic impedance, making it unsuitable for sensor use [40]. Humidity sensors based on PVA polymer have already been proposed, but the results of those studies demonstrated low sensitivity and slow transient response to changes in humidity levels [35,41,42]. To reduce the film’s impedance, thereby enhancing its humidity sensitivity, combining it with another material is essential to address this problem. The electrical characteristics of sensing devices can be improved by depositing 2D material nanoparticles onto this polymer-based film.

Herein, for the first time, we demonstrate a unique sorption layer (multi-layered graphene nanostructures known as graphene flower deposited over PVA film), used to fabricate a fast, highly responsive, and extremely sensitive humidity sensor for future applications in printed electronics. GF material has attractive properties such as a large surface area of 500–2500 m^2^/g, transparent nature, remarkable conductivity, and highly crystalline structure [43,44,45,46,47]. As a result, graphene flower can be considered a viable choice for humidity sensing applications. Screen printing was used to fabricate interdigital electrodes (IDEs), and spin-coating was used to coat an active sensing layer of PVA polymer, followed by the spray-coated deposition of GF nanostructures on these IDEs. Ultra-fast response and recovery time of 2 s and 3.5 s, respectively, were exhibited by the fabricated sensor. The fabricated sensor showed a high sensitivity response of 29 nF/%RH and 15-day stability within the measurable range of 40–90% RH. The PVA/GF-based humidity sensor was also put to the test for a variety of real-time applications, including non-contact humidity detection and oral breathing tests at different rates, the results of which are a part of this study. As a result, the suggested sensor is appropriate for a wide range of other applications because of its cost effectiveness, versatility, and high sensitivity.

## 2. Experimental Methods

### 2.1. Materials and Methods

**Figure 1:**
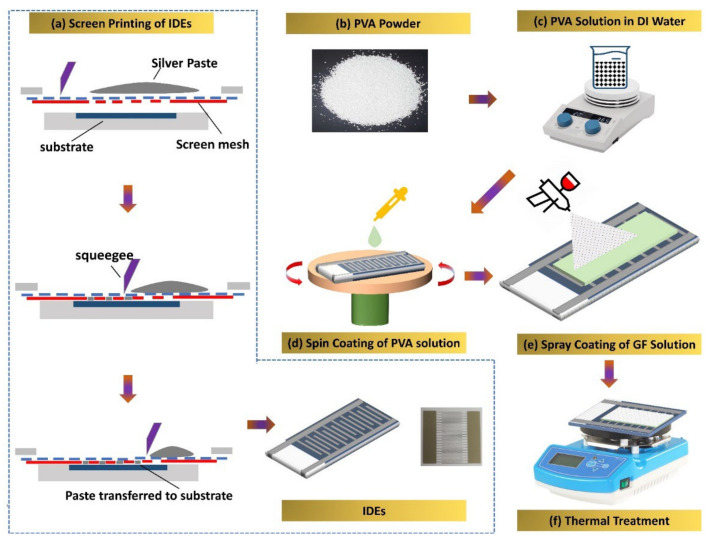
(**a**) Screen printing of Inter Digitated Electrodes over the PET substrate, (**b**) PVA powder, (**c**) PVA solution in the DI water, (**d**) spin coating of PVA solution on electrodes, (**e**) spray coating of graphene flower solution on the PVA-coated IDEs, (**f**) annealing of the active sensing layer of the device.

GF solution in propylene glycol monomethyl ether acetate (PGMEA) (99.9 wt%) was purchased from InALA (Kobe, Japan). PVA polymer was purchased from Sigma-Aldric (Seoul, Korea). For the electrode’s preparation, we purchased screen-printing Ag ink with viscosity 155,000 ± 15,000 cps, density ~2.8 ± 0.2 g/cm^3^, and metal content ~70 ± 2 wt% from InkTec, Ansan-si, Gyeonggi-do, Korea. We purchased the 100-µm-thick PET substrate from AgIC paper. The PVA solution was formed by mixing PVA powder in the DI Water to 1:10 mass ratio and kept at room temperature for 24 h to swell. The solution was then magnetically stirred by keeping it on the hot plate for 12 h at 343 K to dissolve PVA completely as seen in Figure 1c. Graphene flower nanoflake solution in PGMEA solvent was placed on continuous stirring for 30 min to obtain a well-mixed homogeneous solution.

### 2.2. Sensor Fabrication

Figure 1 shows the proposed humidity sensor’s full fabrication process. Flexible PET substrate was first cleaned in ethanol and deionized water then dried at room temperature. Figure 1a shows the detailed fabrication process for patterning IDEs using screen printing (Automax System Engineering AMX-1240M). The electrode fingers were 100 µm wide, and the distance between two IDEs was 200 µm. After fabrication of IDEs, the PVA solution was spin-coated on the IDEs in two steps (500 rpm for 5 s and 1000 rpm for 15 s) to obtain a thin film and subsequently allowed to dry at 30 °C for 24 h, as illustrated in Figure 1d. A solution of GF in PGMEA solvent was spray-coated onto an obtained thin film of PVA that had already been spin-coated, followed by heating the sample at a 50 °C on hot plate for 30 min to let the solvent evaporate as shown in Figure 1e,f. Figure S1 in Supplementary Data depicts the bi-layered sensor’s equivalent circuit.

### 2.3. Characterization

The scanning electron microscope TESCAN MIRA 3 (Brno, Czech Republic) was used to analyze the surface morphology of the deposited active material, while dispersive X-ray (EDS) spectroscopy was used to investigate the elemental composition. The crystal structure of GF and its composite with PVA was studied using an X-ray diffractometer (Panalytical X’PERT PRO, Malvern, UK). Fourier transform infrared (FTIR) spectroscopy was performed by using Fourier Transform infrared spectrometer ALPHA II (Bruker, Billerica, MA, USA). Additionally, Raman spectroscopy was performed using Micro Raman Spectrometer System LABRAM HR EV (Horiba, Kyoto, Japan).

### 2.4. Sensor Evaluation

**Figure 2:**
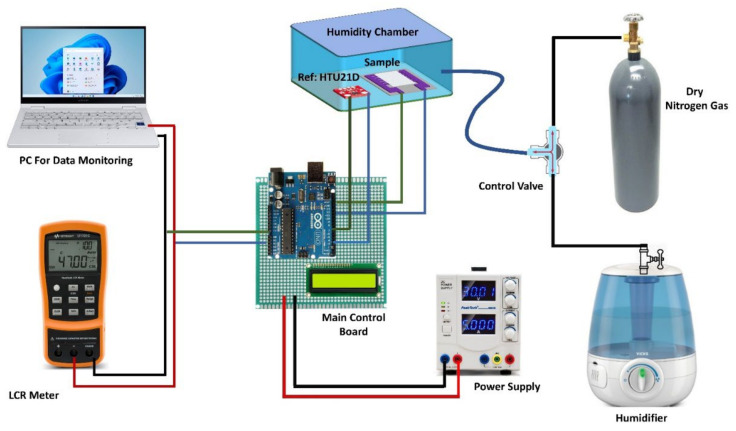
Custom made Experimental setup used to perform all Electrical characterizations.

We studied the electrical response of the sensor by exposing it to varying levels of RH over a wide range. Figure 2 shows a customized data collection and conversion unit, for recording the output of the sensor, the HTU-21D reference sensor along with an Arduino board and a digital display was employed to determine the actual level of RH within the customized measurement chamber and provide feedback to keep it at the desired level. The testing chamber comprised outlet and inlet valves for regulating the flow of dry nitrogen and humid air gases. Dry nitrogen gas was introduced into the testing chamber while humid air was evacuated from the chamber exit to reduce humidity until the RH reached a set testing value and vice versa for increasing humidity level inside the chamber. In the sealed measurement chamber, the constructed sensor was put beside the commercial humidity sensor for data recording. We employed a power supply, and using a Keysight LCR meter U1733C (Santa Rosa, CA, USA), digital parameters such as capacitance and impedance responses were acquired. For both capacitance and impedance response measurements, the LCR meter provides a 0.74 V_rms_ source alternating voltage signal. When the RH of the chamber changed the capacitance and impedance spectra incorporating the impedance and capacitance responses were acquired by interfacing the LCR meter with a personal computer. The proposed sensor’s humidity responses were recorded over a wide humidity range, and the temperature was kept constant at 22 °C during the experiment. The test frequency was maintained constant at 1 kHz and 10 kHz while examining the sensor’s electrical response. The humidification level of the chamber was gradually increased while recording the measurement to get the maximum number of data points.

## 3. Results and Discussion

**Figure 3:**
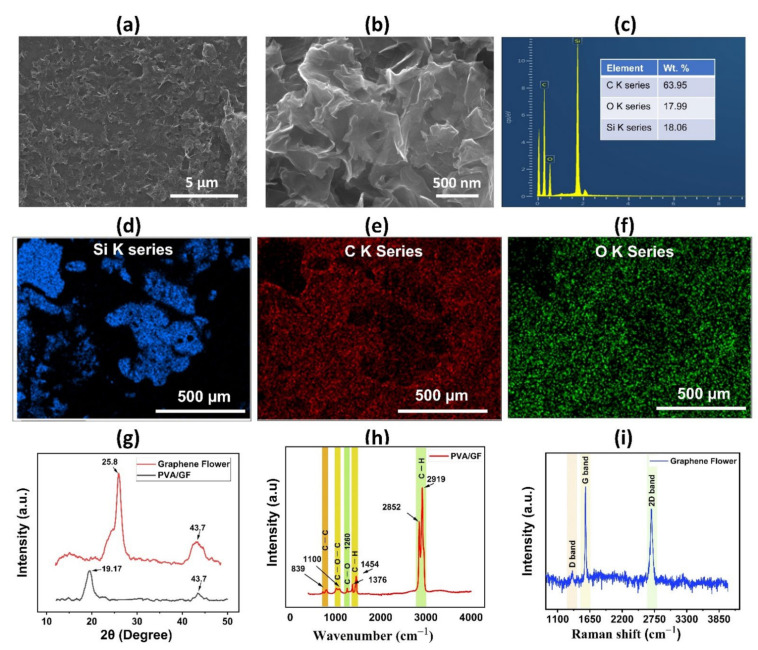
Sensor characterization (**a**) SEM image of the PVA/graphene flower composite thin film with resolution levels of 5 µm. (**b**) SEM image of the PVA/graphene flower composite thin film with resolution levels of 500 nm. (**c**) EDS analysis of composite film. (**d**) EDS layered image. (**e**) O K series. (**f**) C K series of composite materials. (**g**) XRD analysis results of graphene flower, and PVA/graphene flower composite thin film. (**h**) Fourier transform infrared spectroscopy (FTIR) to characterize the composite layer materials. (**i**) Raman spectra of a graphene flower.

To examine the surface morphology of the proposed humidity sensor’s PVA/GF composite thin film, a scanning electron microscope image was taken of the active layer, as shown in Figure 3a,b, which shows the uniform dispersion of active material over the substrate at resolution levels of 5 µm and 500 nm, respectively. The cross-sectional image of the PVA film is shown in Figure S2, displaying its thickness of 2.32 µm. EDS analysis is typically used to confirm the elemental compositions of a given sample; this measurement was conducted on the active sensing layer at a resolution level of 500 µm. As shown in Figure 3c, the EDS images reveal carbon (63.95%) and oxygen (17.99%), and silicon (18.06%), respectively, while O K, Si K, and C K series are shown in Figure 3d–f. The existence of a PVA/GF bilayer thin coating was confirmed by EDS mapping. The crystalline structure of GF and PVA thin films was investigated using an X-ray diffractometer (Empyrean) with a step size of 0.02° from 10° to 50°. The characteristic peaks of PVA/GF composite at 19.5° are due to the semi-crystalline structure of the PVA membrane. The semi-crystalline structure of PVA is held together by intramolecular and intermolecular hydrogen bonding. The peak at 25.8° (around 2*θ* = 27°), in Figure 3g, shows that the material contains the carbon-based elemental composition of graphene flower and internal carbon atoms or a molecular structure. The XRD pattern also exhibits a weak (100) peak at 43.2°, suggesting that (100) planes perpendicular to the sample plane are preferred. The wide peak in the graphene flower at 2*θ* = 25.8° disappears in the composites as the graphene flower is dispersed into the PVA matrix, implying graphene disorder and loss of structural regularity and intercalation of PVA into inter-layers of graphene. As a result, the graphene nanostructures are thought to have been disseminated into the PVA matrix on a molecular level. In the composites, similar results were reported for studies performed previously, by adding graphene nanostructures in the PVA with different volume ratios. To determine the purity of the materials and study their probable interaction, Fourier transform infrared spectroscopy (FTIR) was used to investigate the functional groups in active layer materials, as shown in Figure 3h. FTIR spectrum shows the absorption peaks at of C=H peak that represents the presence of GF in the composite in the range of 2921 and 2849 cm^−1^ originating from C–H stretching vibrations. From the FTIR spectrum of PVA, peaks at 1450 cm^−1^ (C–H bending) and1100 cm^−1^ (C–O–C stretching) can be observed, confirming the presence of pure PVA in the composite. Small peaks positioned at 1376 cm^−1^ can be attributed to C–H bonds, while at 1260 is specific to C–O bonds, and a small peak at 839 cm^−1^ is attributed to C–C stretching vibration. Chemical composition of the sensor film was determined using Raman spectroscopy (LabRAM HR Raman). Figure 3i depicts the Raman spectroscopy analysis of GF indicating the G, D and 2D peaks, we can deduce that GF has multiple layers. The peak bands for the GF peak were observed at 1352 cm^−1^, 1575 cm^−1^, and 2696 cm^−1^, respectively. The 3D Nano Profiling System (NV2200) was used to find out the surface roughness of sensing active material. From this 3D view, the thickness of PVA/GF, and GF was estimated to be 4.7 µm, and 2.1 µm respectively as shown in Figures S3 and S4.

**Figure 4:**
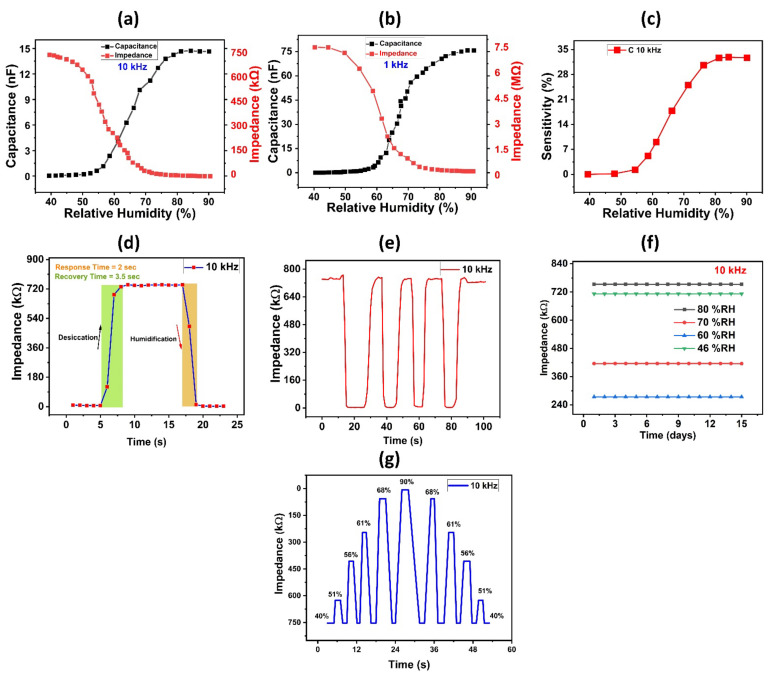
(**a**) Capacitance and impedance response of the proposed sensor at 10 kHz. (**b**) Capacitance and impedance response of the proposed sensor at 1 kHz. (**c**) Sensitivity response of proposed humidity sensor. (**d**) Response and recovery time graph. (**e**) Reproducibility of humidity sensor over various cycles of humidification and desiccation. (**f**) Stability results of humidity sensor. (**g**) Impedance responses of PVA/GF composite under switching RH.

We measured the impedance and capacitance responses under different humidity levels at test frequencies of 1 kHz, and 10 kHz to explore electrical performance of the proposed humidity sensor. Figure 4a,b showed the investigated impedance and capacitance responses. As shown in Figure 4b, impedance varied from 7.3 MΩ to 0.2 MΩ with a change in RH from 40% to 90% at the test frequency of 1 kHz. When relative humidity is increased, the sheet impedance of the deposited thin film drops, resulting in a decrease in impedance. Similarly, at 10 kHz frequency, the impedance starts at 757 kΩ at 40% relative humidity and then falls linearly with increasing relative humidity until it reaches 8.9 kΩ at 90% RH, as seen in Figure 4a. In addition, at experimental frequencies of 10 kHz and 1 kHz, the capacitance responses of the fabricated humidity sensor were also measured against changes in relative humidity. When the relative humidity rises, the absorption of water molecules into the surface of the active sensing layer also rises, leading to changes in the dielectric coefficient and, as a result, a shift in the capacitance response of a sensor. Figure 4b depicts the measured values of capacitance variation at a test frequency of 1 kHz. With a change in RH from 40% to 90%, capacitance increased from 0.04 nF to 75.6 nF at 1 kHz frequency. Similarly, at a test frequency of 10 kHz, the capacitance response starts from 0.0446 nF at 40% RH and rises to 75.6 nF at 90% RH, as shown in Figure 4a. Supplementary Data, Figure S5 shows comparison graph values of capacitance of 3 test frequencies. Sensor’s response recorded separately on GF and PVA for comparison with the composite layer’s sensitivity is shown in Supplementary Data Figures S6–S9, respectively. We clearly see, the improvement of sensitivity values as depicted in Figure S10. Additionally, the sensor’s sensitivity response was determined using Formula (1).

(1) $$S_{\;x}=\frac{C_{RH}-C_{RH_{o}}}{C_{RH_{o}}\;}\times 100\%$$

*C_RH_* and *C_RHo_* are the capacitances at %RH and 40% RH, respectively. Figure 4c depicts the relationship between the sensor’s sensitivity response and capacitance at a frequency of 10 kHz. The experimental value rises linearly from 0.00% to 32,680.26% with an overall variation of 29 nF/%RH, as shown in Figure 4c, indicating that the sensor exhibits extreme sensitivity response to changing humidity levels. We tested our proposed sensor with higher film thickness of PVA film (6.81 µm) as seen in Figure S11a, it was observed that with a change in RH from 40% to 90%, capacitance increased from 2.35 pF to 4.53 pF at 10 kHz frequency. Also, as shown in Figure S11b, impedance varied linearly from 59.6 MΩ to 42 MΩ with a change in RH from 40% to 90% at the test frequency of 10 kHz. The dependence of sensitivity on film thickness was observed, a very small sensitivity of 0 to 92.6% was shown in Supplementary Data of Figure S11c. The overall sensitivity response of the proposed sensor was calculated using the following formula:

(2) $$\mathrm{Sc}=\frac{C_{max}-C_{min}}{RH_{max}-RH_{o}\;}\times 100\%\;$$

where Sc represents the overall variation of the sensitivity response. Figure 4d depicts the transient response data of the sensor. To measure the response time of the sensor, humidity level was increased from 40% RH to 90% RH, and subsequently, humidity level was decreased back to 40% to measure the recovery time of the sensor. The proposed humidity sensor’s rapid recovery time was found to be reliable. Figure 4d shows that the sensor demonstrated a robust transient performance with a response time of 2 s and a recovery time of 3.5 s for its impedance response at 10 kHz, which are considered to be suitable values for various applications requiring quick recovery and response times. Sensor’s transient response at 1 kHz frequency is also shown in the Supplementary Data Figure S5. Table 1 provides a comparison of our humidity sensor’s sensing properties with those of other previously published sensors. Table 1 shows that, when compared to other published humidity sensors, our suggested scheme has exceptional detection parameters and fast response. Figure 4e shows the dynamic response parameters of the PVA/GF sensor at 40–90% RH. When the RH returns to its initial condition, the impendence of the resultant PVA/GF sensor recovers to its original value, and the sensitivity response of our sample does not change over numerous cycles, showing that the sample has strong repeatability. Another essential characteristic to consider when evaluating a sensor’s efficiency is its stability; hence, we tested the stability of our developed humidity sensor by keeping it in several fixed humidity conditions for 15 days, including 46, 70, 62, and 80% RH levels, and measuring impedance at a test frequency of 10 kHz. The suggested sensor was highly stable at each RH level, as shown in Figure 4f. Impedance response of the PVA/GF-based sensor at various RH percent levels is shown in Figure 4g. Figure S12 shows zoomed portion of varying Impedance responses of PVA/GF composite under switching RH. The sensor’s reversibility and reliability were investigated through response/recovery cycles, the sensor was exposed from low %RH to high %RH, and then from high %RH to low %RH. Hysteresis characteristic graph is shown in Figures S13 and S14 of Supplementary Data. As seen a very small hysteresis was observed.

The capacity of PVA to sense moisture is due to an OH group, which can be seen in the PVA structure. The capacity of GF to identify water molecules is dependent mainly upon the high specific surface area percentages and hydrophilic functional groups, including such hydroxyl and carboxyl groups, linked to its surface. Additional adsorption sites, such as voids and flaws, are created when PVA is mixed into GF layers. They can be formed at the interface between the two nanomaterials, which helps to increase humidity sensing. Moreover, GF has a low electrical resistance and a high concentration of charge carrier. It can act as an anchor to enhance electron transport in metallic nanoparticles, which helps to modify PVA. When the sensor is exposed to varying humidity levels in this study, it easily interacts with the water vapor, causing the dielectric constant to fluctuate. As per the findings of the research, GF nanostructures without PVA have a lack of stability and reproducibility, which is a stumbling barrier for sensor applications. PVA will absorb or desorb water molecules in a humid atmosphere, resulting in a change in the dielectric constant. The dielectric constant will increase as the humidity level rises, electrical response, i.e., capacitance and impedance, will change as the dielectric constant changes. When humidity rises, there will be more hydrogen bonding between water vapor and the OH groups in PVA. The quantity of H^+^ ions will rise under these conditions, and they will be able to hop between water vapor molecules (Grotthuss mechanism). When the amount of water vapor in the atmosphere increases, more H^+^ ions migrate, causing the PVA/GF conductivity to rise. Protons are the primary carriers responsible for electrical conductivity when exposed to moisture.

**Figure 5:**
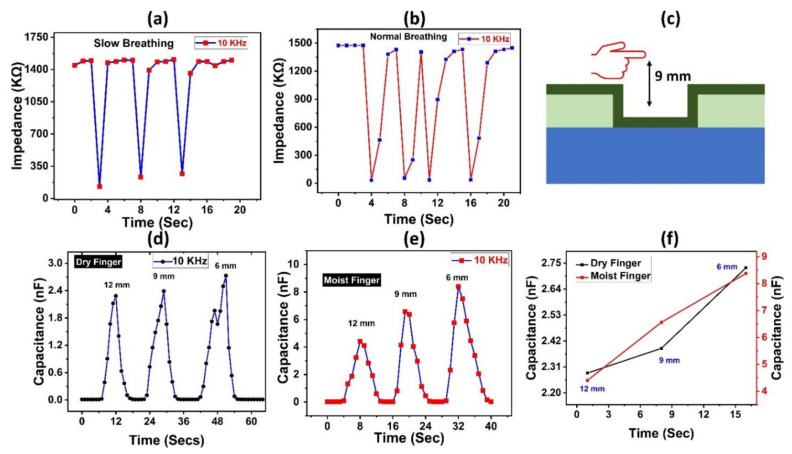
Impedance response to (**a**) human slow breathing and (**b**) human normal oral breathing. (**c**) Schematic diagram of oral breathing test of the humidity sensor. (**d**) Capacitance curve for the proximity test for the dry finger of the proposed sensor at fixed distances of 6 mm, 9 mm, and 12 mm. (**e**) Capacitance curve for the proximity test for the moist finger of the proposed sensor at fixed distances of 6 mm, 9 mm, and 12 mm. (**f**) Capacitance measurements of sensor at different proximities.

The suggested sensor was evaluated for various applications depending on its outstanding sensing capability, including non-contact humidity sensing of the fingertip. Furthermore, we put our sample to the test for human healthcare monitoring applications by introducing it to human respiration to detect water vapors. Our sensor quickly responded to and detected minor %RH fluctuations. Figure 5b depicts the recorded data points for our suggested sensor’s open-air exhale and open-air inhale tests compared to normal respiration. In Figure 5a for slow breathing rate, during exhale, the impedance plunged significantly from 1450 kΩ towards 130 kΩ, then quickly returned to its previous value within a few seconds. Similarly, Figure 5b shows similar changes in impedance values for the normal human breathing rate. Water vapors enable the conductivity of the sensor layer to fluctuate when inhaling and exhaling the air. The observed capacitance shift is in the nano-farad region, indicating great promise in healthcare monitoring applications. Figure 5c shows a schematic representation of non-contact humidity detection for humidity using an adult fingertip at distances of 6 mm, 9 mm, and 12 mm. Because a dry finger also retains some moisture, capacitance ranges from 0.02 nF to 2.735 nF, as seen in Figure 5d. The capacitance of a wet finger grew from 0.09 nF to 8.3772 nF at a frequency of 10 kHz, as illustrated in Figure 5e. Figure 5f shows the capacitance values for both dry and wet fingers at different proximity levels. As a result of the above findings, we can determine that our suggested sensor reacts to humidity with no significant inaccuracy.

## 4. Conclusions

In summary, this paper proposed a highly sensitive, fast, and stable humidity sensor based on 2D graphene nanoflakes, with PVA/GF nanocomposite film as the sensing layer. The spray-coated GF solution has a high electrical conductivity and homogeneous dispersion. The suggested humidity detecting device showed a humidity sensing range of 40–90% RH, with the response and recovery periods of 2 s and 3.5 s, respectively, at room temperature. The developed humidity sensor exhibited a capacitance sensitivity response of 29 nF/%RH at 10 kHz with a good linearity and reproducibility. Our proposed device also exhibited different applications, such as contactless skin humidity monitoring, due to its superior sensing performance characteristics. Similarly, we tested our sensor in an open environment and found it to be stable. The humidity detecting performance of the constructed sensor was outstanding. Based on these findings, we anticipate that humidity sensors based on PVA/GF nanocomposite will have a bright future in the field of real-time humidity detecting electronic devices.

## Figures and Tables

**Table 1:** Comparison of the response and recovery times with different humidity sensing materials described.

Sensing Material	RH Range	Response Time	Recovery Time	Sensitivity Response	Sensing Principle	Ref.
Tin(IV)Oxide/Reduced Graphene Oxide	11–97	102 s	6 s	-	-	[48]
Black Phosphorous	11–97	255 s	10 s	-	Resistive	[49]
Graphene-Polystyrene Sulfonic Sodium	30–95	3 s	22 s	-	Impedance	[3]
MWCNT/HEC	20–80	11 s	35 s	0.0485/%RH	Resistive	[50]
PVA/dGO	40–100	10 min	10 min	13.25 MΩ	-	[51]
PVA/graphene nanofibers	10–80	11 s	50 s	66.4%	Resistive	[42]
Au-PVA	0–70	-	-	5.4 nm/%RH	-	[52]
Single-walled CNTs	15–98	290 s	510 s	246.9%	Resistive	[53]
G/ZnO	0–85	1 s	2 s	11.5% (ΔZ/Z)	Impedance	[54]
MWCNT	10–90	3.5 s	5.8 s	55% (ΔR/R)	Resistive	[55]
GR	33–95	5.88 s	6.25 s	33% (ΔI/I)	Current	[56]
MWCNT/HEC/PVPP	10–90	0.8 s	0.78 s	1480% (ΔC/C)	Capacitance	[57]
Fe_2_O_3_	0–100	1.79 s	4.97 s	882% (ΔC/C)	Capacitance	[58]
Ti_3_C_2_/Ag	35–95	0.08 s	0.12 s	125% (ΔC/C)	Capacitance	[59]
PVA/GF	40–90	2 s	3.2 s	29 nF/%RH	Impedance	this work

## Data Availability

The data presented in this study are available on request from the corresponding author.

## Supplementary Materials

The following supporting information can be downloaded at: https://www.mdpi.com/article/10.3390/nano12061026/s1. Figure S1:Equivalent circuit of the bi-layered sensor on IDT electrodes; Figure S2: Cross-sectional image of the PVA film on the SiO_2_ substrate; Figure S3: 3D Nano Profiling image of GF; Figure S4: 3D Nano Profiling image of PVA/GF; Figure S5: Comparison of impedance response at different test frequencies; Figure S6: Impedance and Capacitance-based responses of GF alone as sensing layer at 1 kHz; Figure S7: Impedance and Capacitance-based responses of GF alone as sensing layer at 10 kHz; Figure S8: Impedance and Capacitance-based responses of PVA alone as sensing layer at 10 kHz; Figure S9: Impedance and Capacitance-based responses of PVA alone as sensing layer at 1 kHz; Figure S10: A comparison of sensitivity values Sensors fabricated with PVA and GF as separate layers alone; Figure S11: (a) Cross-sectional image of the thick PVA film on the SiO_2_ substrate, Impedance and Capacitance-based responses (b) and Sensitivity (c) of thick bi-layered PVA/GF as sensing layer at 10 kHz; Figure S12: Impedance responses of PVA/GF composite under switching RH; Figure S13: Hysteresis curve of adsorption and desorption behavior towards relative humidity at 10 kHz; Figure S14: Hysteresis curve of adsorption and desorption behavior towards relative humidity at at 1 kHz.
