# Supplementary material for: Highly Sensitive and Stable Humidity Sensor Based on the Bi-Layered PVA/Graphene Flower Composite Film
Source: Nanomaterials (Basel). 2022 Mar 21;12(6):1026. doi: 10.3390/nano12061026 (PMC8955666; doi:10.3390/nano12061026)
Supplement: Supplementary file 1 [file nanomaterials-12-01026-s001.zip › nanomaterials-1574212-supplementary.pdf]

Supporting Information

Highly Sensitive and Stable Humidity Sensor Based on the Bi-Layered PVA/Graphene Flower Composite Film

Sheik Abdur Rahman †, Shenawar Ali Khan †, Muhammad Muqeet Rehman and Woo-Young Kim *

Faculty of Applied Energy System, Major of Electronic Engineering, Jeju National University, Jeju 63243, Korea; abdurrahman@jejunu.ac.kr (S.A.R.); shenawaralikhan@jejunu.ac.kr (S.A.K.); muqeet1988@jejunu.ac.kr (M.M.R.)
* Correspondence: semigumi@jejunu.ac.kr
† These authors contributed equally to this work.

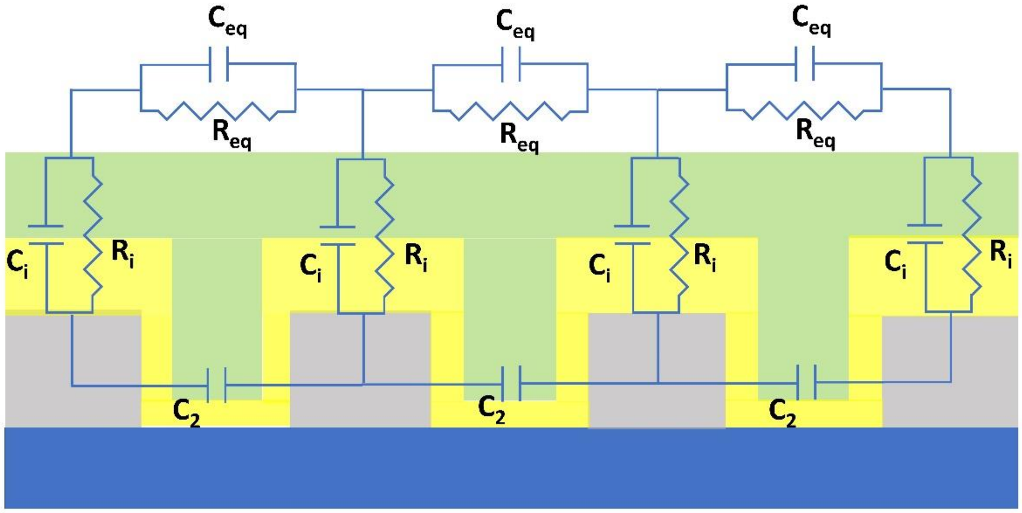

Figure S1. (a) Equivalent circuit of the bi-layered sensor on IDT electrodes.

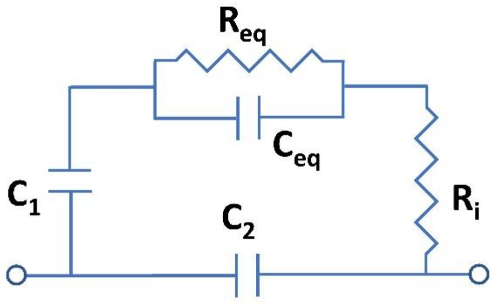

Figure S1. (b) Equivalent circuit of the bi-layered sensor on IDT electrodes.

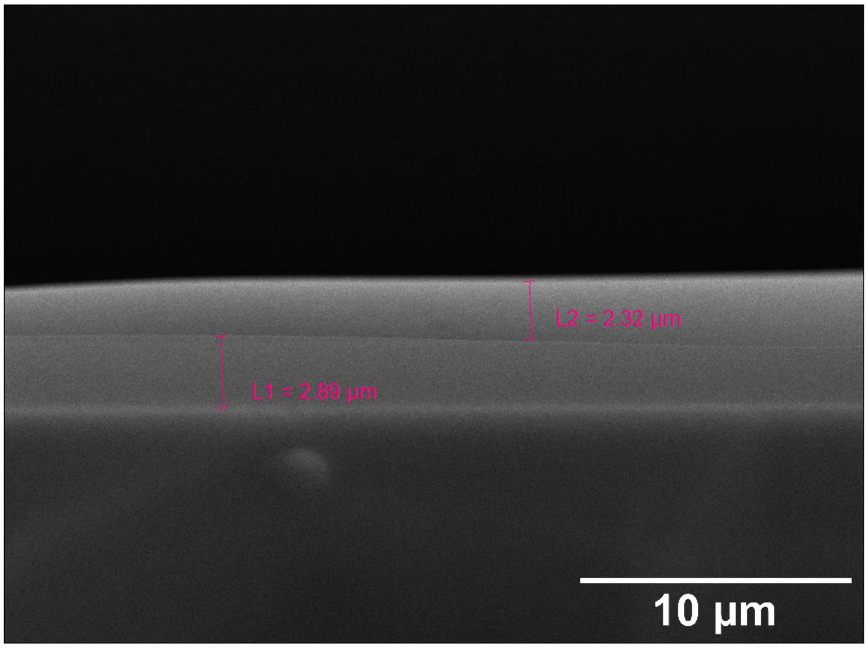

Figure S2. Cross-sectional image of the PVA film on the SiO_2 substrate.

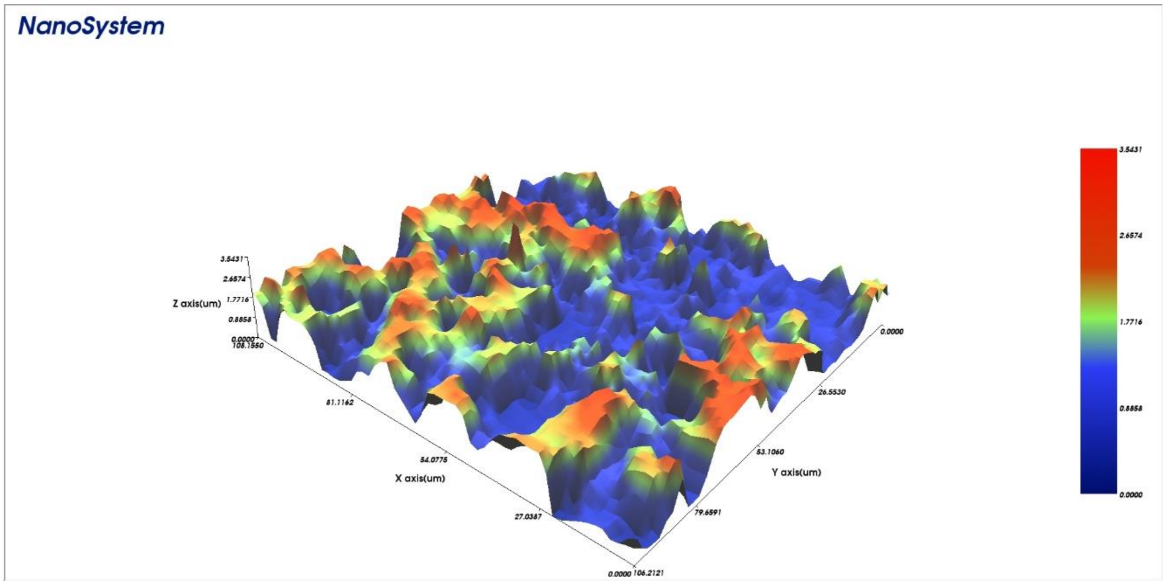

Figure S3. 3D Nano Profiling image of GF

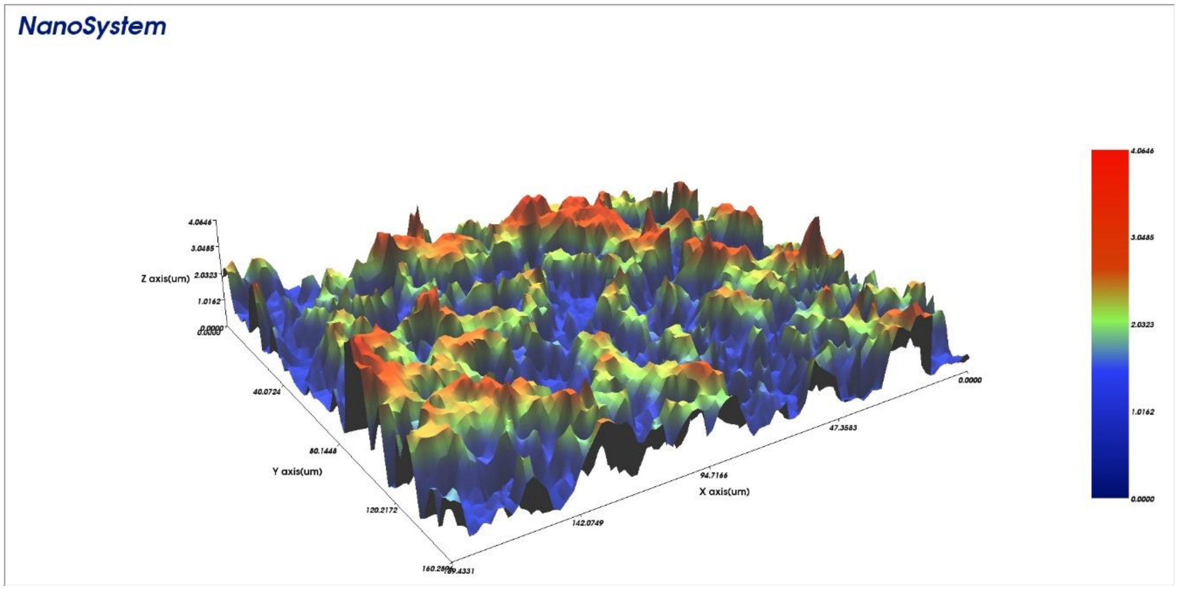

Figure S4. 3D Nano Profiling image of PVA/GF

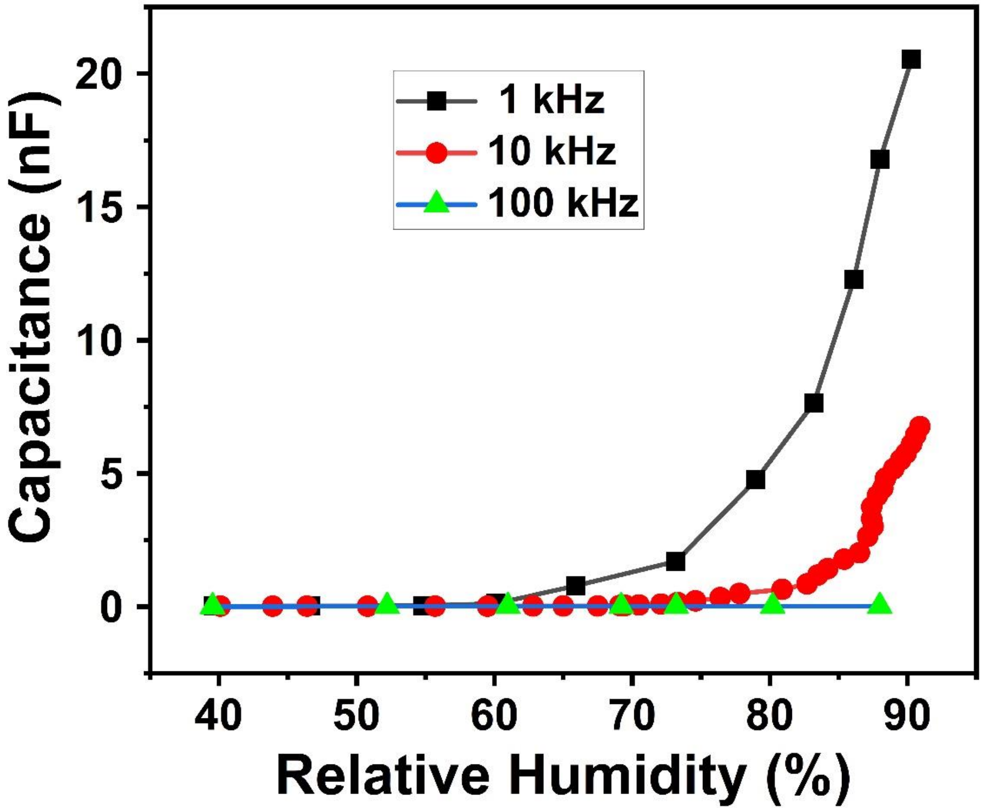

Figure S5. Comparison of impedance response at different test frequencies

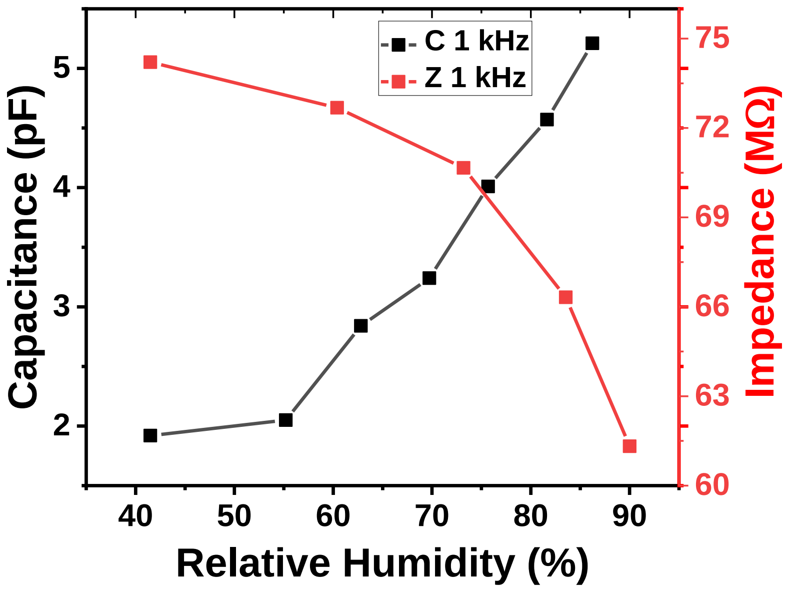

Figure S6. Impedance and Capacitance-based responses of GF alone as sensing layer at 1 kHz

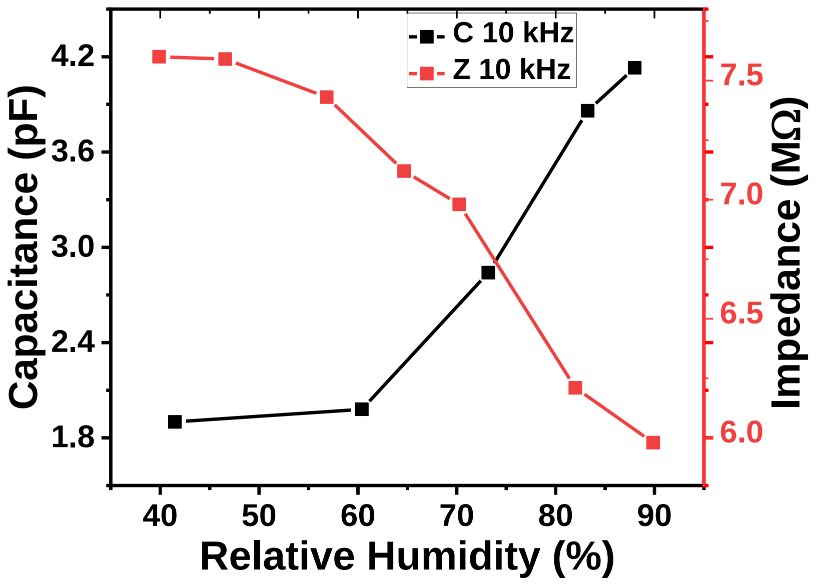

Figure S7. Impedance and Capacitance-based responses of GF alone as sensing layer at 10 kHz

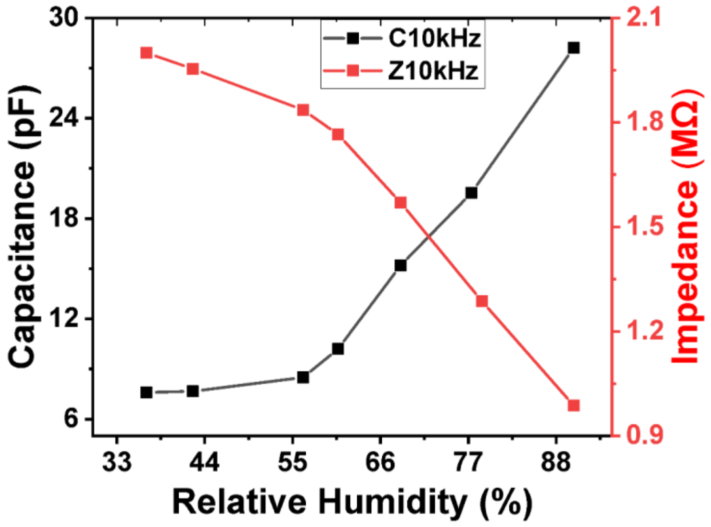

Figure S8. Impedance and Capacitance-based responses of PVA alone as sensing layer at 10 kHz

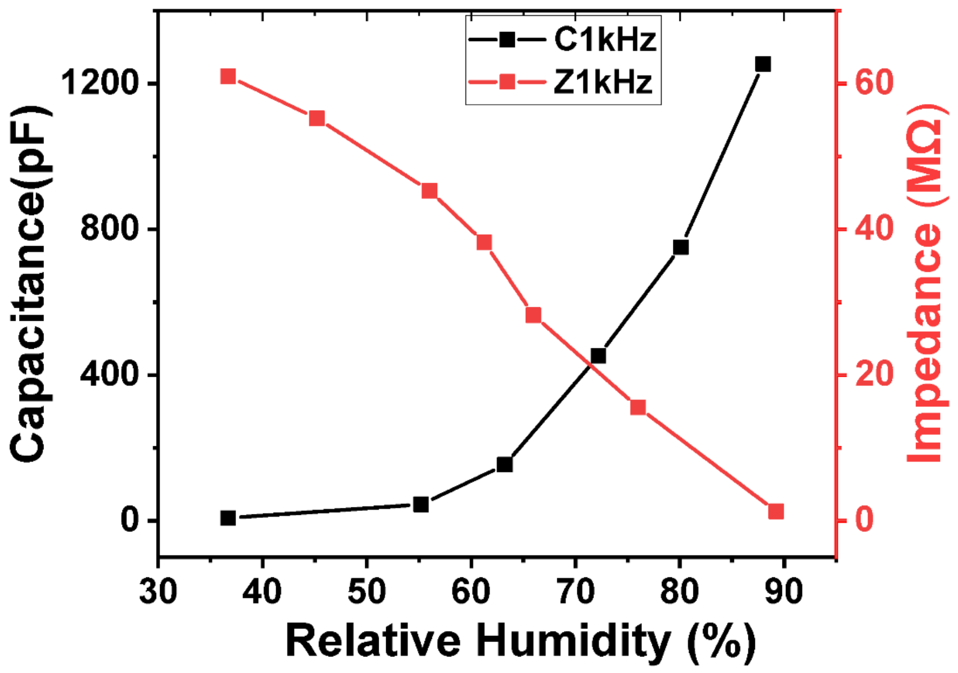

Figure S9. Impedance and Capacitance-based responses of PVA alone as sensing layer at 1 kHz

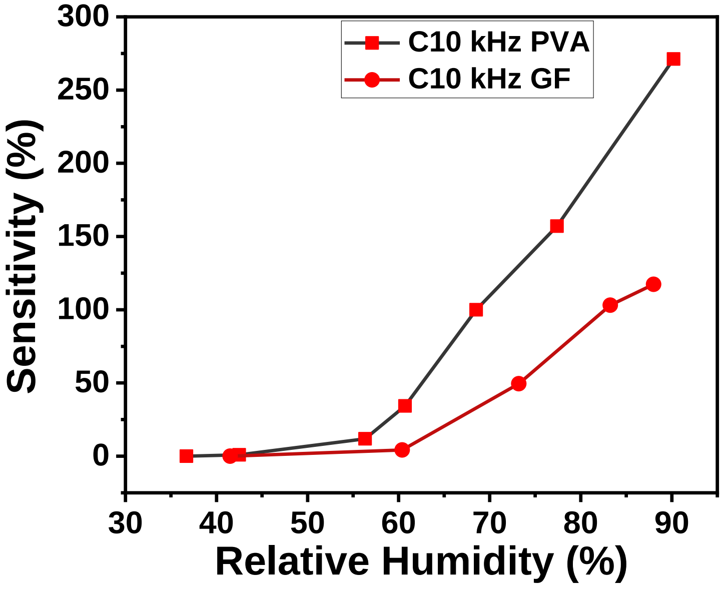

Figure S10. A comparison of sensitivity values Sensors fabricated with PVA and GF as separate layers alone

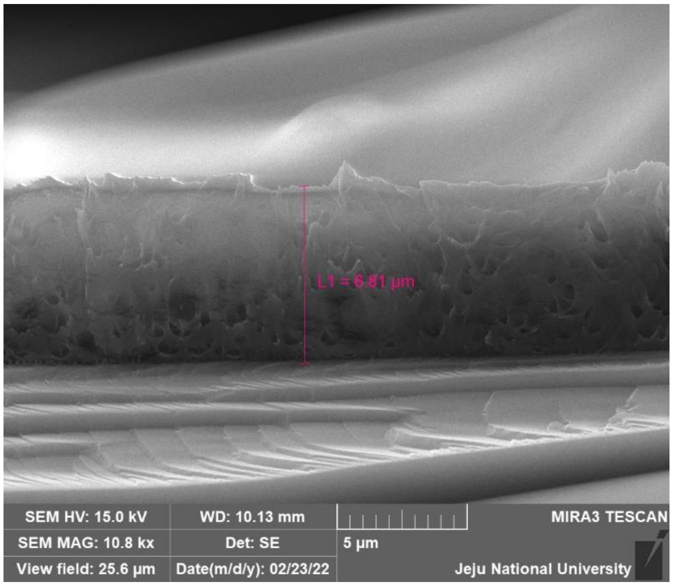

Figure S11. (a) Cross-sectional image of the thick PVA film on the SiO_2 substrate.

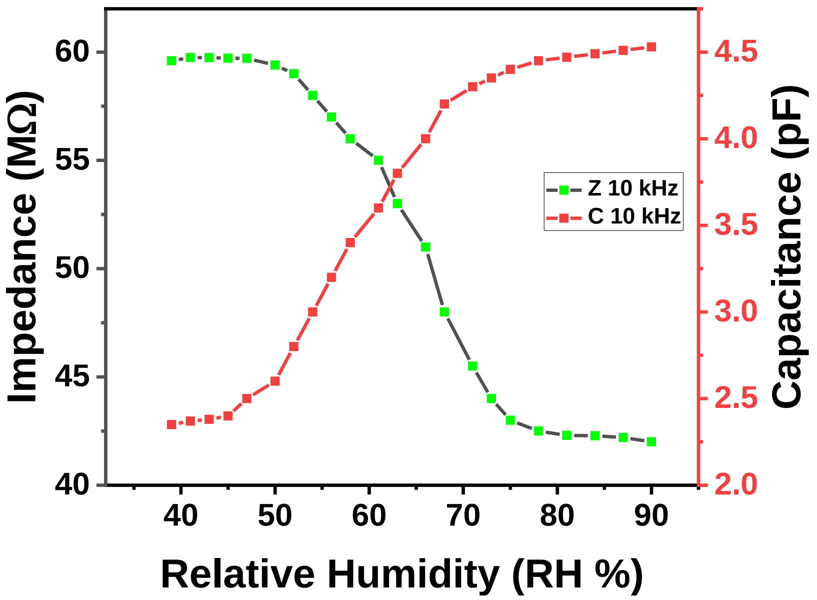

Figure S11. (b) Impedance and Capacitance-based responses of thick bi-layered PVA/GF as sensing layer at 10 kHz

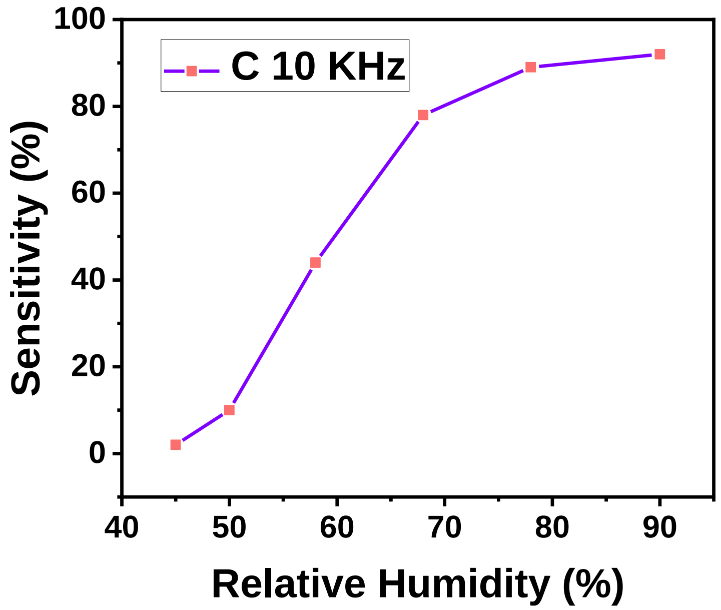

Figure S11. (c) Sensitivity of proposed of thick bi-layered PVA/GF as sensing layer at 10 kHz

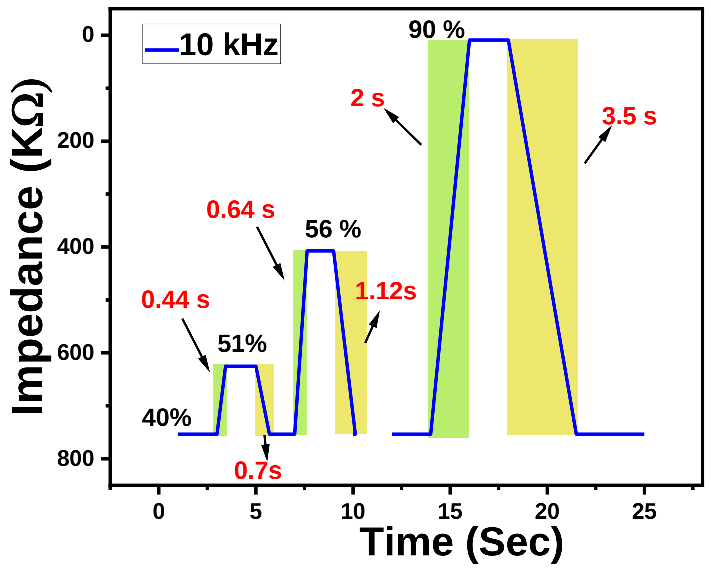

Figure S12. Impedance responses of PVA/GF composite under switching RH

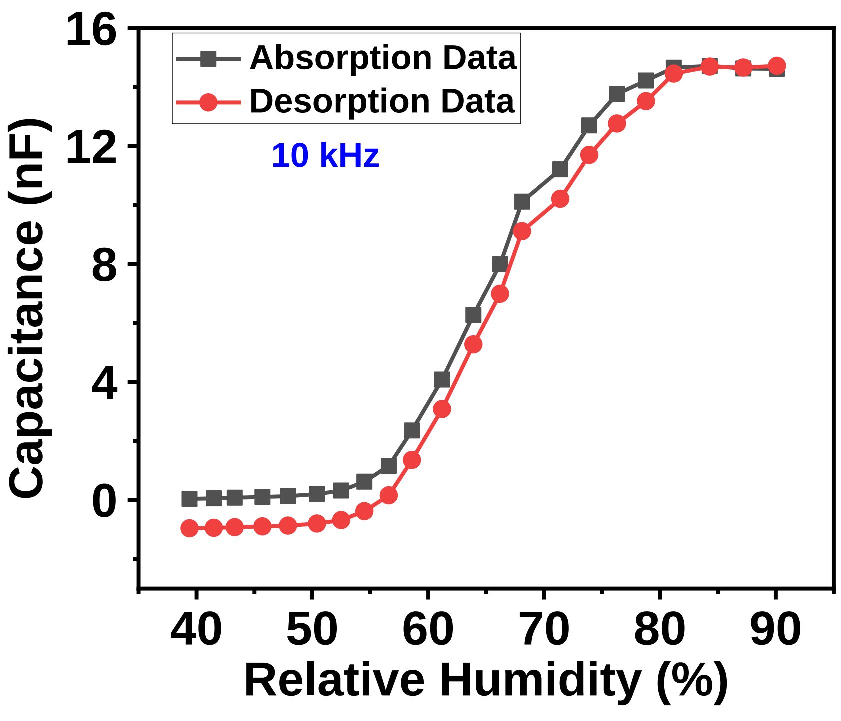

Figure S13. Hysteresis curve of adsorption and desorption behavior towards relative humidity at 10 kHz

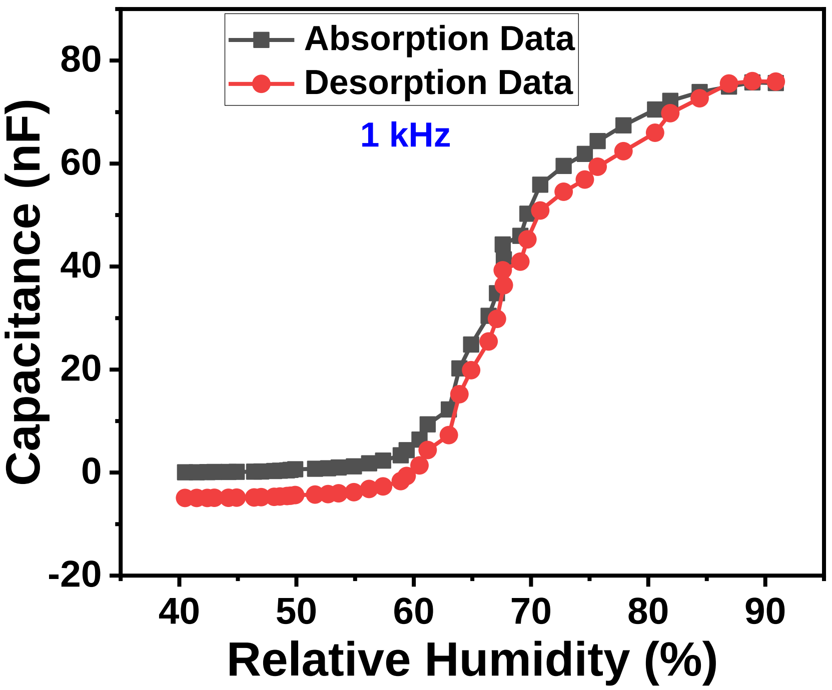

Figure S14. Hysteresis curve of adsorption and desorption behavior towards relative humidity at 1 kHz
